# Cortico‐cortical connectivity within ferret auditory cortex

**DOI:** 10.1002/cne.23784

**Published:** 2015-05-12

**Authors:** Jennifer K. Bizley, Victoria M. Bajo, Fernando R. Nodal, Andrew J. King

**Affiliations:** ^1^ Department of PhysiologyAnatomy and Genetics, University of OxfordOxfordOX1 3PTUnited Kingdom; ^2^ Ear Institute, University College LondonLondonWC1X 8EEUnited Kingdom

**Keywords:** tract tracing, topography, auditory system, carnivore, multisensory

## Abstract

Despite numerous studies of auditory cortical processing in the ferret (*Mustela putorius*), very little is known about the connections between the different regions of the auditory cortex that have been characterized cytoarchitectonically and physiologically. We examined the distribution of retrograde and anterograde labeling after injecting tracers into one or more regions of ferret auditory cortex. Injections of different tracers at frequency‐matched locations in the core areas, the primary auditory cortex (A1) and anterior auditory field (AAF), of the same animal revealed the presence of reciprocal connections with overlapping projections to and from discrete regions within the posterior pseudosylvian and suprasylvian fields (PPF and PSF), suggesting that these connections are frequency specific. In contrast, projections from the primary areas to the anterior dorsal field (ADF) on the anterior ectosylvian gyrus were scattered and non‐overlapping, consistent with the non‐tonotopic organization of this field. The relative strength of the projections originating in each of the primary fields differed, with A1 predominantly targeting the posterior bank fields PPF and PSF, which in turn project to the ventral posterior field, whereas AAF projects more heavily to the ADF, which then projects to the anteroventral field and the pseudosylvian sulcal cortex. These findings suggest that parallel anterior and posterior processing networks may exist, although the connections between different areas often overlap and interactions were present at all levels. J. Comp. Neurol. 523:2187–2210, 2015. © 2015 Wiley Periodicals, Inc.

**Abbreviations**
A1: primary auditory cortex
AAF: anterior auditory field
ADF: anterior dorsal field
AEG: anterior ectosylvian gyrus
aPSSC: anterior posterior bank of the pseudosylvian sulcal cortex
AVF: anterior ventral field
BDA: biotinylated dextran amine (tracer)
CTB: cholera toxin, subunit B (tracer)
EG: ectosylvian gyrus
FR: Fluoro Ruby (tracer)
MGBm: medial division of the MGB
MGB: medial geniculate body
MGBd: dorsal division of the medial geniculate body
MGBv: ventral division of the medical geniculate body
MEG: middle ectosylvian gyrus
pPSSC: posterior bank of the pseudosylvian sulcal cortex
PEG: posterior ectosylvian gyrus
PPF: posterior pseudosylvian field
PSF: posterior suprasylvian field
Pss: pseudosylvian sulcus
SSY: suprasylvian sulcal (area)
Sss: suprasylvian sulcus
VP: ventral posterior (area)
WM: white matter

The ferret is now one of the most widely used animal models for studying auditory cortical processing and plasticity (reviewed by Nodal and King, 2014). The presence of multiple auditory cortical areas on the ectosylvian gyrus (EG) of this species was first demonstrated by using 2‐deoxyglucose autoradiography (Wallace et al., 1997) and subsequently confirmed by using optical imaging of intrinsic signals (Nelken et al., 2004) and single‐unit recording (Kelly et al., 1986; Kelly and Judge, 1994; Kowalski et al., 1995; Bizley et al., 2005). Although most electrophysiological recording studies have focused on the primary auditory cortex (A1) (Phillips et al., 1988; Kowalski et al., 1996; Schnupp et al., 2001; Fritz et al., 2003; Rabinowitz et al., 2011; Keating et al., 2013), the nonprimary auditory fields in this species are now receiving increasing attention (Nelken et al., 2008; Bizley et al., 2009, 2010, 2013; Walker et al., 2011; Atiani et al., 2014).

In contrast to the growing number of investigations into the physiological properties of cortical fields that lie beyond the auditory core, little attention has been paid to the anatomical organization of the nonprimary cortex in ferrets. By placing tracer deposits in the middle ectosylvian gyrus (MEG), previous studies have demonstrated the topography of the inputs from the medial geniculate nucleus (Pallas et al., 1990), and the presence of connections within and between this region and both the anterior and posterior parts of the gyrus (AEG and PEG, respectively) (Wallace and Bajwa, 1991; Gao and Pallas, 1999). These studies have shown that regions on the posterior bank are more strongly connected with the MEG than those on the anterior bank (Pallas and Sur, 1993). Several studies have looked at connections between specific fields located on the ectosylvian gyrus (EG) and other sensory cortices (Ramsay and Meredith, 2004; Manger et al., 2005; Bizley et al., 2007a; Keniston et al., 2009), and at the descending, subcortical connections that originate in the auditory cortex (Bajo et al., 2007, 2010a, b). A comprehensive anatomical investigation of cortico‐cortical connectivity within the EG of this species has not, however, been carried out. This information is vital for interpreting the data obtained from both electrophysiological recordings and imaging studies, as well as the behavioral consequences of deactivating specific regions of the auditory cortex, and for relating these findings to those described in other species.

In this study, we investigate the cortico‐cortical connections of the different fields that make up the ferret auditory cortex. By making single or multiple injections of tracers into physiologically defined regions of these fields, and then examining the resulting retrograde and, where appropriate, anterograde labeling, we were able to build up a picture of the pattern of connectivity both within and between the acoustically responsive areas located on the ferret EG.

## MATERIALS AND METHODS

All the experiments were approved by the local ethical review committee at the University of Oxford and authorized by the UK Home Office. Thirteen healthy adult ferrets (both male and female, aged > 4 months) were used in the study.

### Injections and tracers

Single and multiple injections of neural tracers were made into different parts of the auditory cortex (Table 1). Each animal received one, two or, in a single instance, three, separate injections in the auditory cortex. The tracers used were 10% dextran tetramethylrhodamine (lysine fixable, 3,000 and 10,000 MW, Fluoro Ruby [FR]; Molecular Probes, Eugene, OR), 10% dextran biotin fixable (BDA, 10,000 MW; Molecular Probes), and 1% cholera toxin B (CTB, List Biological Laboratories, Campbell, CA). The size of the resulting injection sites and their diffusion halos varied depending on the tracer used (for example, FR tended to diffuse to produce a much larger halo than BDA) even with the same injection parameters. The use of different tracers avoided the limitations associated with any individual tracer, and allowed us to combine more than one tracer in the same animal. Full details of each injection site, including the injection site depth and volume, are detailed in Table 1.

**Table 1:** Injection Sites^a^

Animal			CF		IS size (mm^3^)	Depth (µm)	IS center
no.	Tracer	IS location	(kHz)	Plane	Center (halo)	of center (range)	(layers)
b **F0532**	FR	A1	20	Coronal	0.22 (3.09)	0.64 (0.28–0.90)	III–VI
	BDA	AAF	20		0.04	0.45 (0–0.9)	I–V
**F0522**	FR	large A1/AAF		Coronal	0.46 (22.02)	0.47 (0–0.96)	I–VI
**F0536**	BDA	A1/PSF	2	Coronal	0.05 (0.39)	1.06 (0.95–1.37)	V‐VI
	FR	A1	19		<0.01	1,100	V
**F0252**	FR	A1/AAF	15	flattened	0.07 (1.02)	0.5 (0.05‐–.95)	I–VI
	BDA	A1	1		0.18 (1.39)	0.48 (0.05–0.90)	I–VI
b **F0268**	BDA	AAF	7	flattened	0.05 (0.22)	0.6 (0.1–1.3)	I–VI
	FR	A1	7		0.04 (1.2)	0.6 (0.1–1.3)	I–VI
	CTB	A1/AAF	7		0.01 (0.19)	0.65 (0.1–1.4)	I–VI
**F0404**	BDA	AAF	7	flattened	0.04 (0.68)	0.8 (0.1–1.5)	I–VI
	CTB	A1	7		0.004 (0.2)	1.05 (0.55–1.6)	I–VI
**F0535**	FR	AVF	Noise	Coronal	0.14 (7.20)	0.8 (0.4–1.3)	III–VI
	BDA	ADF	10		0.01	1.1 (0.7–1.4)	IV–VI
b **F0523**	FR	AVF	LED, noise	Coronal	0.71 (1.68)	0.55 (0 −1.1)	I–VI
	BDA	ADF	<2, broad		0.03 (0.39)	0.5 (0–1)	I–VI
b **F0533**	BDA	VP	1	Coronal	0.01 (0.13)	0.3 (0–0.4)	I–III
	CTB	PPF	8		0.039 (1.58)	0.7 (0–1.4)	I–V
b **F0717**	BDA	PSF		Coronal	0.36 (1.2)	0.65 (0–1.3)	I–VI
**F0510**	CTB	AVF		Coronal	1.29 (7.87)	0.75 (0–1.5)	I–VI
b **F0504**	BDA	PPF		Flattened	0.54 (6.09)	0.6 (0.1–1.1)	I–VI
b **F0505**	BDA	AEG/aPSSC		Flattened	1.31 (7.13)	0.8 (0.1–1.7)	I–VI
	CTB	PPF			(0.63)	0.7 (0.05–1.65)	I–VI

a The locations (cortical field) of the injections site (IS) for each animal and tracer are listed. When physiological recordings were obtained, the characteristic frequency (CF) is listed. Also detailed is the plane of sectioning (coronal or flattened) and the injection site volume (core and halo measurements are provided) along with the depth of the injection site center and the span of the core (note that in all cases except for F0536 and the BDA injection in F0533 the halo spanned all layers).

b Cases illustrated within the article. Ferret numbers are also shown within each figure.

For abbreviations, see list.

**Table 2:** Primary Antibodies Used

Antigen	Immunogen	Source, host species, cat#, clone or lot#, RRID	Concentration used
Anti‐CTB	Anti‐cholera toxin B	List, goat, cat# A6397, RRID:AB_2313636	1:15,000
Anti‐FR	Anti‐tetramethylrhodamine	Molecular Probes, rabbit polyclonal, cat# A6397, RRID:AB_10375968	1:6,000
SMI^32^	Neurofilament heavy chain (nonphosphorylated)	Sternberger Monoclonals, mouse monoclonal, cat# SMI‐32P, RRID: AB_231492	1:4,000

After sedation with Domitor (1 ml/kg body weight (BW), i.m. of medetomidine hydrochloride; Pfizer, Kent, UK), anesthesia was induced with Saffan (2 ml/kg BW of alfaxalone/alfadolone acetate, i.m.; Schering‐Plough Animal Health, Welwyn Garden City, UK) and maintained with an intravenous infusion of a mixture of Domitor (0.022 mg/kg BW/h) and Ketaset (5 ml/kg BW/h; ketamine hydrochloride; Fort Dodge Animal Health, Southampton, UK) in a saline solution. Dexadreson (0.5 mg/kg BW/h of dexamethasone; Intervet UK, Milton Keynes, UK) and Atrocare (0.006 mg/kg BW/h of atropine sulfhate; Animalcare, York, UK) were added to the infusate to avoid cerebral edema and minimize secretions in the respiratory tract, respectively. The electrocardiogram was monitored and body temperature was maintained at ∼38°C throughout the experimental procedure.

The animal was mounted in a stereotaxic frame fitted with hollow ear bars to facilitate acoustic stimulus presentation. A midline incision was made in the scalp, and the left temporal muscle was retracted to expose the skull. After local application of Marcaine (bupivacaine, Astra Pharmaceuticals, Kings Langley, UK), the left EG was exposed by a craniotomy, and the dura was removed. Tracer injections were, where possible (17/23 injection sites; see Table 1), made in physiologically identified cortical regions. When physiological verification was not possible, the locations of the tracer injections were targeted and assigned to a particular cortical field based on our previous descriptions of ferret auditory cortex (Bizley et al., 2005) and were subsequently confirmed cytoarchitectonically (see below).

Because there is no available stereotaxic atlas for the ferret brain, and neither bregma nor lambda are visible in adult ferrets, we targeted our craniotomies by using measurements made relative to the temporal ridge (12 mm ventral) and the occipital ridge (11 mm anterior). These coordinates defined the dorsocaudal corner of a square whose size varied from 4 × 5 mm (anterior–posterior × dorsal–ventral to expose only the MEG) to ∼6 × 8 mm (to expose the AEG and/or PEG also). Such craniotomies allowed us to visualize the dorsal tip of the suprasylvian sulcus (i.e., the A1) and by then visualizing the locations of both the suprasylvian and pseudosylvian sulci we were able to target recordings and/or injections to specific cortical regions.

Electrophysiological recordings were made by using single tungsten‐in‐glass electrodes with stimulus presentation and data acquisition performed by using TDT system 3 hardware (Tucker Davis Technologies, Alachua, FL) and BrainWare software (London, UK). Signals were amplified and digitized for off‐line analysis. Acoustic stimuli were noise bursts and pure tones of varying frequency (150 Hz to 20 kHz) and intensity, as in Bizley et al. (2005).

For tracer injections, a glass micropipette was lowered into the brain and BDA, FR, or CTB was injected, in most cases, by iontophoresis using a positive current of 5 µA and a half‐duty cycle of 7 seconds for a duration of 15 minutes. In a small number of cases, FR and CTB were injected by pressure with a nanoejector (Nanoject II; Drummond Scientific, Broomall, PA). Depths were chosen based either on the electrophysiological recordings or on the known cortical thickness of the area to be injected, but were typically at 900 µm from the cortical surface. Our goal was to fill an entire cortical column and we therefore targeted layers III–V. Once the injections were complete, the micropipette was left in place for 10 minutes before being withdrawn, the dura mater was lifted back in place, and the piece of cranium that had previously been removed was replaced. The temporal muscle was repositioned over the skull and attached to adjacent musculature, and the scalp margins were sutured together. The animals received perioperative and subsequent postoperative analgesia with Vetergesic (0.05 ml of buprenorphine hydrochloride, i.m.; Alstoe Animal Health, Melton Mowbray, UK). Details of the tracers used in each cortical region, the number of injections and, when measured, the frequency preference of multiunit activity at the injection site are given in Table 1.

### Histological analysis

Transcardial perfusion was performed 2–5 weeks after tracer injection following a terminal overdose with Euthatal (2 ml of 200 mg/ml of pentobarbital sodium; Merial Animal Health, Harlow, UK). The blood vessels were flushed with 300 ml of 0.9% saline followed by 1 liter of fresh 4% paraformaldehyde in 0.1 M phosphate buffer (PB), at pH 7.4. The brain was dissected from the skull, maintained in the same fixative for several hours, and immersed in a 30% sucrose solution in 0.1 M PB for 3 days. In five cases, the two hemispheres were dissected and placed between two glass slides to be maintained flat in the sucrose solution. In those cases, the flattened cortex was later sectioned in the tangential plane and the brainstem in the coronal plane; the other eight brains were sectioned in standard coronal plane (Table 1). Then 50‐μm sections were cut on a freezing microtome, and six sets of serial sections were collected in 0.1 M PB. Every third section was used to analyze the tracer labeling.

FR and CTB were visualized with immunohistochemistry reactions, whereas BDA was reacted only with avidin biotin peroxidase (Vectastain Elite ABC Kit; Vector, Burlingame, CA). Sections were washed several times in 10 mM phosphate‐buffered saline (PBS) with 0.1% Triton X100 (PBS‐Tx) and incubated overnight at 4°C in the primary antibody (FR: anti‐tetramethylrhodamine, rabbit immunoglobulin G [IgG]; Molecular Probes; Life Technologies cat# A6397, RRID:AB_10375968, dilution 1:6,000; CTB: goat‐anti‐CTB, dilution 1:15,000, List Biological cat# 104, RRID:AB_2313636). After washing 3 times in PBS‐Tx, sections were incubated for 2 hours in the biotinylated secondary antibody (biotinylated goat anti‐rabbit IgG H + L [FR] Vector, cat# BA‐1000, RRID:AB_2313606 or rabbit‐anti‐goat [CTB], dilution 1:200; Vector,cat# BA‐5000, RRID:AB_2336126) at room temperature. Sections were once again washed and incubated for 90 minutes in avidin biotin peroxidase, washed in PBS, and then incubated with the chromogen solution, 3,3′‐diamino‐ benzidine (DAB; Sigma‐Aldrich, Dorset, UK). Sections were incubated in 0.4 mM DAB and 9.14 mM H_2_O_2_ in 0.1 M PB until the reaction product was visualized. When BDA and FR or CTB were injected in the same animal, the BDA was first visualized with ABC followed by DAB enhanced with 2.53 mM nickel ammonium sulfate. The second tracer (FR or CTB) was subsequently visualized by using the appropriate protocol with DAB only as the chromogen. Reactions were stopped by rinsing the sections several times in 0.1 M PB. Sections were mounted on gelatinized glass slides, air dried, dehydrated, and coverslipped.

For every case, one set of serial sections (one every 300 μm) was counterstained with 0.2% cresyl violet, another set was selected to visualize cytochrome oxidase (CO) activity, and a third set was used to perform SMI_32_ immunohistochemistry. CO staining was obtained after 12 hours of incubation with 4% sucrose, 0.025% cytochrome C (Sigma‐Aldrich), and 0.05% DAB in 0.1 M PB at 37°C. To stain neurofilament H in neurons, we used a monoclonal mouse anti‐SMI_32_ (dilution 1:4,000; Sternberg Monoclonals, Latherville, MA; mouse monoclonal, cat# SMI‐32P, RRID: AB_231492). After immersion for 60 minutes in a blocking serum solution with 5% normal horse serum, the sections were incubated overnight at 5°C with the mouse antibody and 2% normal horse serum in 10 mM PBS. Mouse biotinylated secondary antibody was used after brief washings in 10 mM PBS (biotinylated horse anti‐mouse IgG (H + L), Vector, cat# BA‐2000, RRID: AB_2313581, dilution 1:200 in PBS with 2% normal horse serum; Vector). Immunoreaction was followed by several washings in PBS, incubation in ABC, and visualization using DAB with nickel–cobalt intensification.

Histological analysis and drawings were performed with a Leica DMR microscope and a digital Leica camera by using TWAIN software (Leica Microsystems, Heerbrugg, Switzerland). Drawings of labeling and layer and field boundaries from adjacent sections stained for Nissl or immunoreactive for SMI_32_ were scanned and digitized. Images were overlaid within CorelDraw (Corel, Ottawa, ON, Canada) to determine the location of labeled cells and terminal fields, and to produce the resulting figures. Quantification was achieved by counting the numbers of object elements within CorelDraw for all sections in a series for each tracer.

### Ferret auditory cortex

**Figure 1:**
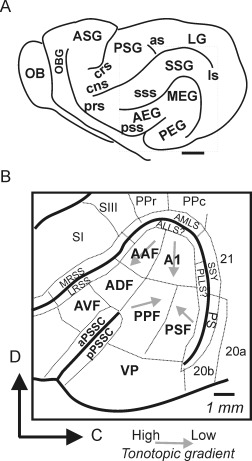
Location of ferret auditory cortex. **A:** Schematic of a whole ferret brain showing the main sulci and gyri. The auditory cortex is located on the ectosylvian gyrus. OB, olfactory bulb; OBG, orbital gyrus; ASG, anterior sygmoid gyrus; PSG, posterior sygmoid gyrus; SSG, syprasylvian gyrus; LG, lateral gyrus; prs, presylvian sulcus; crs, cruciate sulcus; cns, coronal sulcus; as, anseate sulcus; sss, suprasylvian sulcus; pss, pseudosylvian sulcus; ls, lateral sulcus. **B:** Schematic showing the identified sensory areas within and around the ectosylvian gyrus. The auditory areas characterized to date comprise the primary auditory cortex (A1), the anterior auditory field (AAF), the posterior pseudosylvian field (PPF), the posterior suprasylvian field (PSF), the ventral posterior field (VP), and the anterior dorsal field (ADF). Multisensory (anterior ventral field [AVF]; anterior and posterior pseudosylvian sulcal cortex [PSSC]), parietal (rostral and caudal posterior parietal fields [PPr, PPc]), visual (areas 20 and 21, the suprasylvian sulcal fields [SSY], PS, and anteromedial lateral suprasylvian [AMLS]), and somatosensory areas (SI, SIII, and the medial bank of the rostral suprasylvian sulcus [MRSS]) are also shown. D, dorsal; C, caudal. The direction of high–low frequency gradients within tonotopically organized fields is shown with gray arrows. Scale bar = 5 mm in A,B.

Figure 1A shows the organization of the cerebral cortex in the ferret, with the EG (where the auditory cortex is located) enlarged in Figure 1B. Multiple areas have been identified based on their physiological response and anatomical properties. Our goal was to place targeted injections into the six areas characterized electrophysiologically so far, two in each region of the EG and labeled in bold in Figure 1B. In the MEG we targeted the A1 and the anterior auditory field (AAF), which are the tonotopically organized primary or core areas (Kowalski et al., 1995; Wallace et al., 1997; Nelken et al., 2004; Bizley et al., 2005), In the PEG, injections were placed in the two further tonotopic fields identified in ferret auditory cortex: the posterior pseudosylvian and the posterior suprasylvian fields (PPF and PSF). Finally, in the AEG, tracers were placed in the anterior dorsal field (ADF) and the anterior ventral field (AVF), which are not tonotopically organized. In addition to investigating the patterns of connectivity among these six physiologically identified auditory fields, we also considered patterns of labeling among several additional areas. The ventral posterior field (VP) has been identified on the basis of its cortico‐collicular connectivity and cytoarchitecture (Bajo et al., 2007), but has yet to be characterized electrophysiologically. The pseudosylvian sulcal cortex (PSSC) lies within the pseudosylvian sulcus and has been shown to receive inputs from the primary visual and somatosensory cortex (Ramsay and Meredith, 2004). The anterior bank of the pseudosylvian sulcal cortex (aPSSC) projects prominently to the superior colliculus (Bajo et al., 2010a), whereas the posterior bank (pPSSC) has only sparse connectivity. We therefore consider the patterns of connections to the anterior and posterior banks separately. Finally, we document projections to the anterolateral suprasylvian sulcus (ALLS), which lies in the bank of the suprasylvian sulcus (sss) dorsal to the A1 (Homman‐Ludiye et al., 2010).

In all cases, the major subdivisions of the auditory cortex were identified by using SMI_32_ immunoreactivity, and CO and Nissl staining. These methods allowed the boundaries between the MEG, PEG, and AEG to be distinguished (Bajo et al., 2007). The physiologically identified fields within each of these areas are not cytoarchitectonically distinguishable. Therefore subdivisions within the MEG, PEG, and AEG were made according to their known physiological organization (Bizley et al., 2005). On this basis, the caudal two‐thirds of the MEG was classified as the A1, with the AAF occupying the rostral one‐third. The PEG was divided equally into the PPF in the rostral half of the PEG and the PSF in the caudal half. The ADF typically occupies the most dorsal one‐third of the AEG, with the AVF lying ventral to that. When considering labeling in the PSSC, we took into account the whole length of the sulcus but divided it into anterior and posterior banks. The ALLS lies within the suprasylvian sulcus surrounding the MEG, but it is cytoarchitectonically distinct due to the large layer V pyramidal neurons found there (Homman‐Ludiye et al., 2010).

We confirmed the location of all injection sites histologically by reconstructing the full injection site and comparing this with the cytoarchitectonically established boundaries as detailed above by using Neurolucida software (MBF Bioscience, MicroBrightField, Williston, VT). These reconstructions were used to estimate the volume and the depth of each injection site (both its core and halo; Table 1). Additionally, we examined whether labeling was present in the thalamus (including both large and small terminals), inferior colliculus, and contralateral cortex. In 20/23 cases both the reconstruction and the additional labeling in these areas indicated that the injection site had encompassed all cortical layers. In only three injections did the injection halo not extend across all six layers; in case F0533 the injection site in the VP was very superficial, and both injections in F0536 were restricted to infra granular layers.

## RESULTS

Ferret auditory cortex encompasses much of the EG and contains within it six physiologically defined areas, in addition to a number of other cytoarchitectonically or anatomically identified areas. Because the physiologically identified areas differ in their sensitivity to spatial and nonspatial features of a sound source (Bizley and King, 2008; Bizley et al., 2009; Bizley et al., 2010), an open question is the extent to which they represent different processing pathways, and how these areas relate to cortical fields in other species. To address these questions, a series of retrograde and anterograde injections (Table 1) were made in a total of 13 ferrets to examine the projections within and between each of the physiologically or anatomically defined auditory areas.

### Tracer injections in the MEG

**Figure 2:**
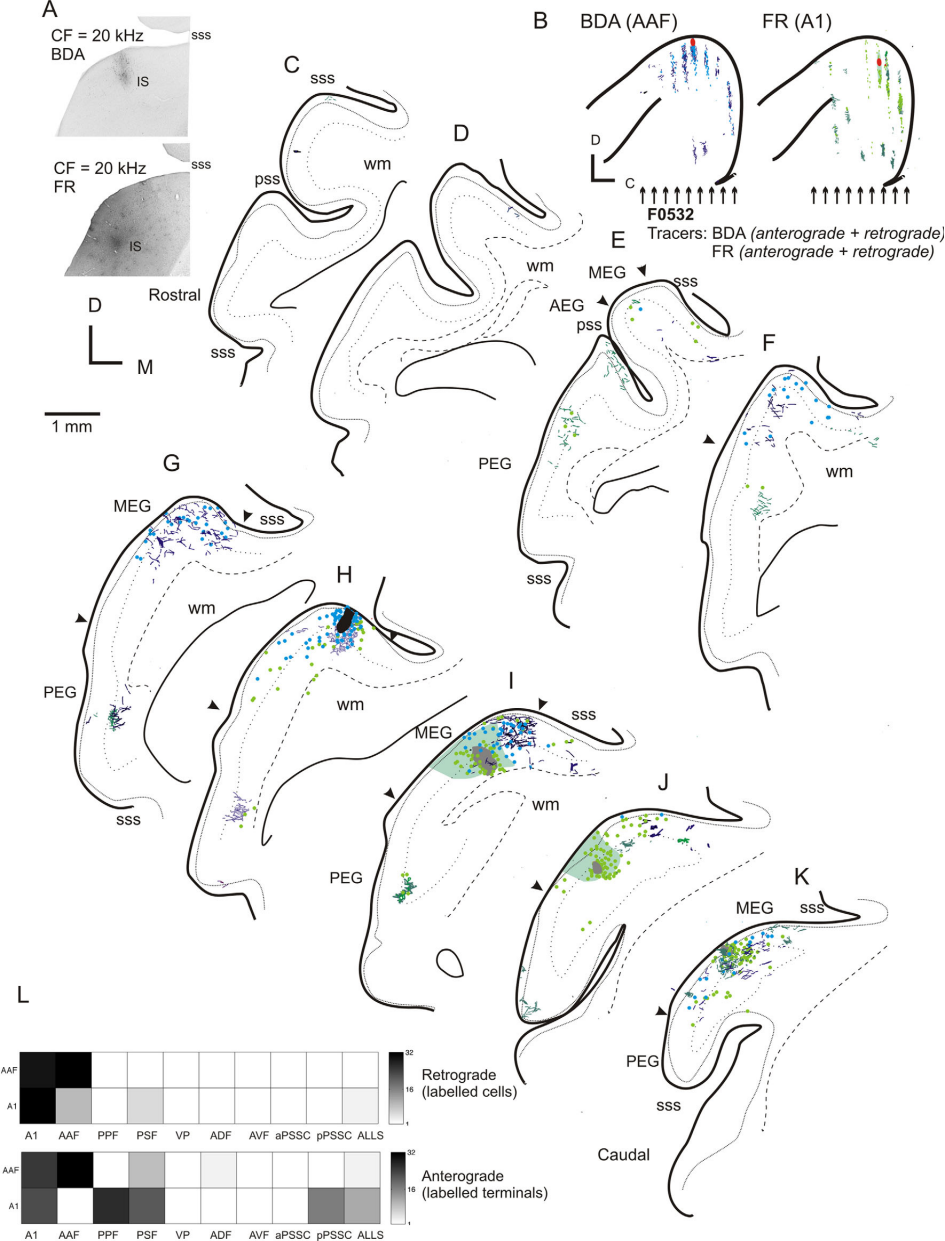
Distribution of anterograde and retrograde labeling in the ipsilateral auditory cortex following frequency‐matched (CF = 20 kHz) injections of BDA and FR into high‐frequency MEG. **A,B:** Photographs (A) and schematics (B) showing the location of the injection sites (marked in red in B) and topology of the resulting label across the coronal sections (locations indicated by arrows in B drawn in C–K). **C–K:** Sections are ordered from the most rostral (C) to the most caudal (K), with the anterograde (BDA, dark blue lines; FR, dark green lines) and retrograde labeling (BDA, light blue circles; FR, light green circles) indicated. The stippled, dotted, and dashed lines in this and subsequent figures represent the boundaries between layers I/II, the location of layer IV, and the white matter, respectively. Sulci and gyri are labeled in alternate sections. The arrows indicate the ventral limits of the MEG determined using SMI_32_ immunohistochemistry. **L:** Quantitative summary of the retrograde and anterograde labeling that results from these injections. For abbreviations, see list. Scale bar = 1 mm on left (applies to A–K).

In three animals we placed multiple tracer injections into frequency‐matched areas of the MEG. This allowed us to directly compare the projection patterns of the A1 and AAF and explore to what extent projections from the MEG were frequency‐specific in nature. Figure 2 shows the pattern of labeling observed after two frequency‐matched (characteristic frequency [CF] = 20 kHz) injections were placed into the high‐frequency A1 and AAF (Fig. 2A,B,H–J, tracers BDA and FR). Labeling from the two injections was overlapping, forming a band of cells and terminals that ran rostrocaudally across the MEG through both injection sites and therefore likely corresponds to an isofrequency lamina (Fig. 2G–K). It is also notable that sparse, scattered, labeled cells were located throughout the MEG (e.g., Fig. 2E,J). The ventral boundary of the MEG (marked by the arrowheads) was determined using SMI_32_ immunoreactivity, and back‐filled cells were present at locations as far ventral as that boundary. Retrogradely labeled cells were found throughout the cortical depth. Back‐filled cells and sparse terminal fields were also evident in the ALLS (Fig. 2H–J).

**Figure 3:**
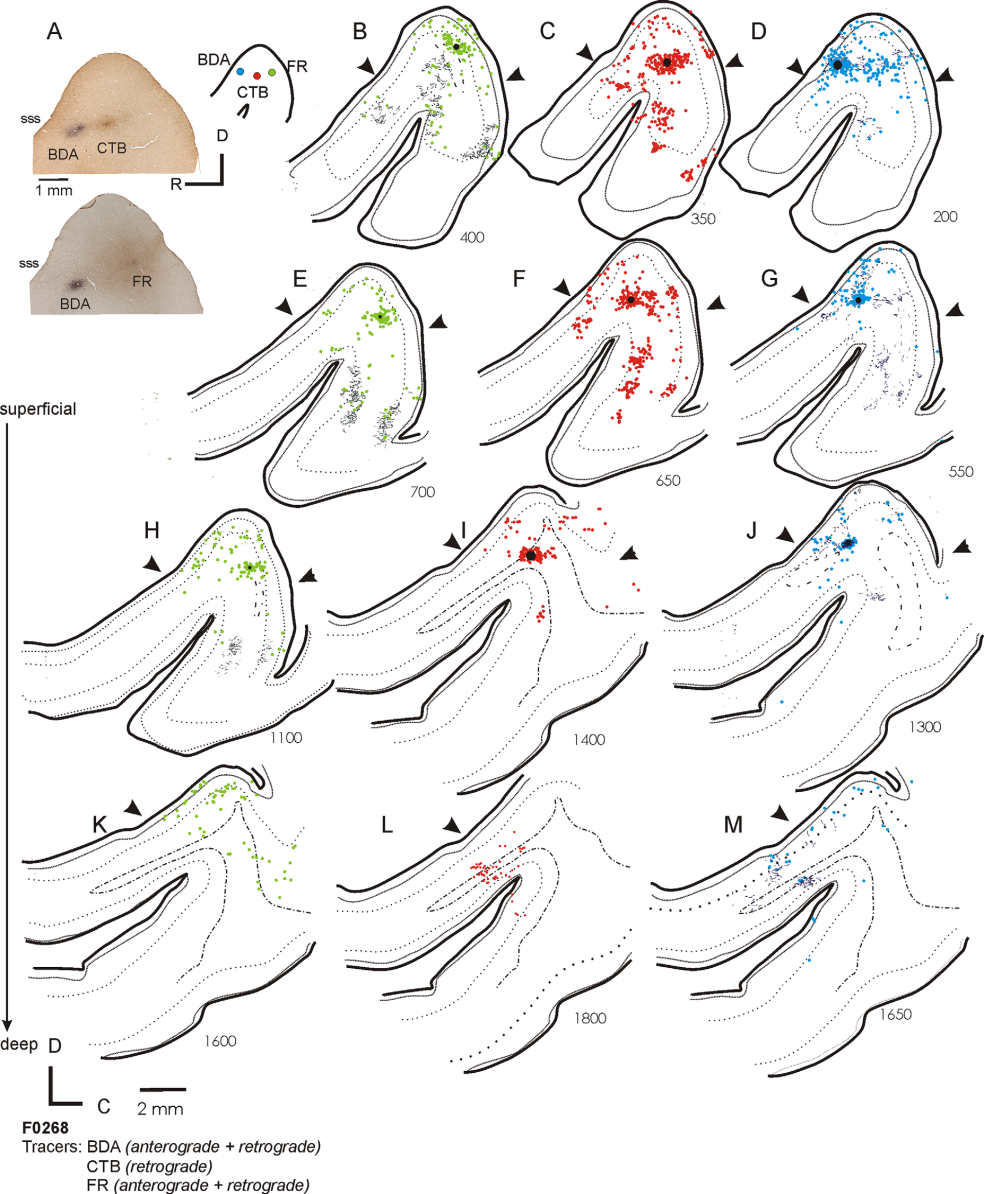
Flattened tangential sections illustrating the distribution of ipsilateral labeling in auditory cortex after three frequency‐matched injections into the MEG. **A:** Photographs showing the location of the three injections and summary diagram (inset). BDA was injected at the most anterior location (blue circle), FR at the most caudal location (green circle), and CTB (red circle) into the center of the MEG. All three injection sites had a CF of 7 kHz. **B–M:** Each row shows sections from a different depth, relative to the flattened cortical surface, with the most superficial sections shown first (B,C,D) and subsequent rows showing progressively deeper sections (exact distances from the pial surface are shown in µm next to each section). Injection sites are indicated by black circles. Each column plots the patterns of label for a different tracer. B, E, H, and K all show anterograde (gray lines) and retrograde (green circles) labeling following the injection of FR into A1. The central column (C,F,I,L) shows retrograde labeling (back‐filled cells are indicated by the red circles) after the injection of CTB. Sections in the right column (D,G,J,M) show anterograde (dark blue lines) and retrograde (light blue circles) labeling following the injection of BDA into AAF. For abbreviations, see list. Scale bar = 1 mm in A; 2 mm in K (applies to B–M).

**Figure 4:**
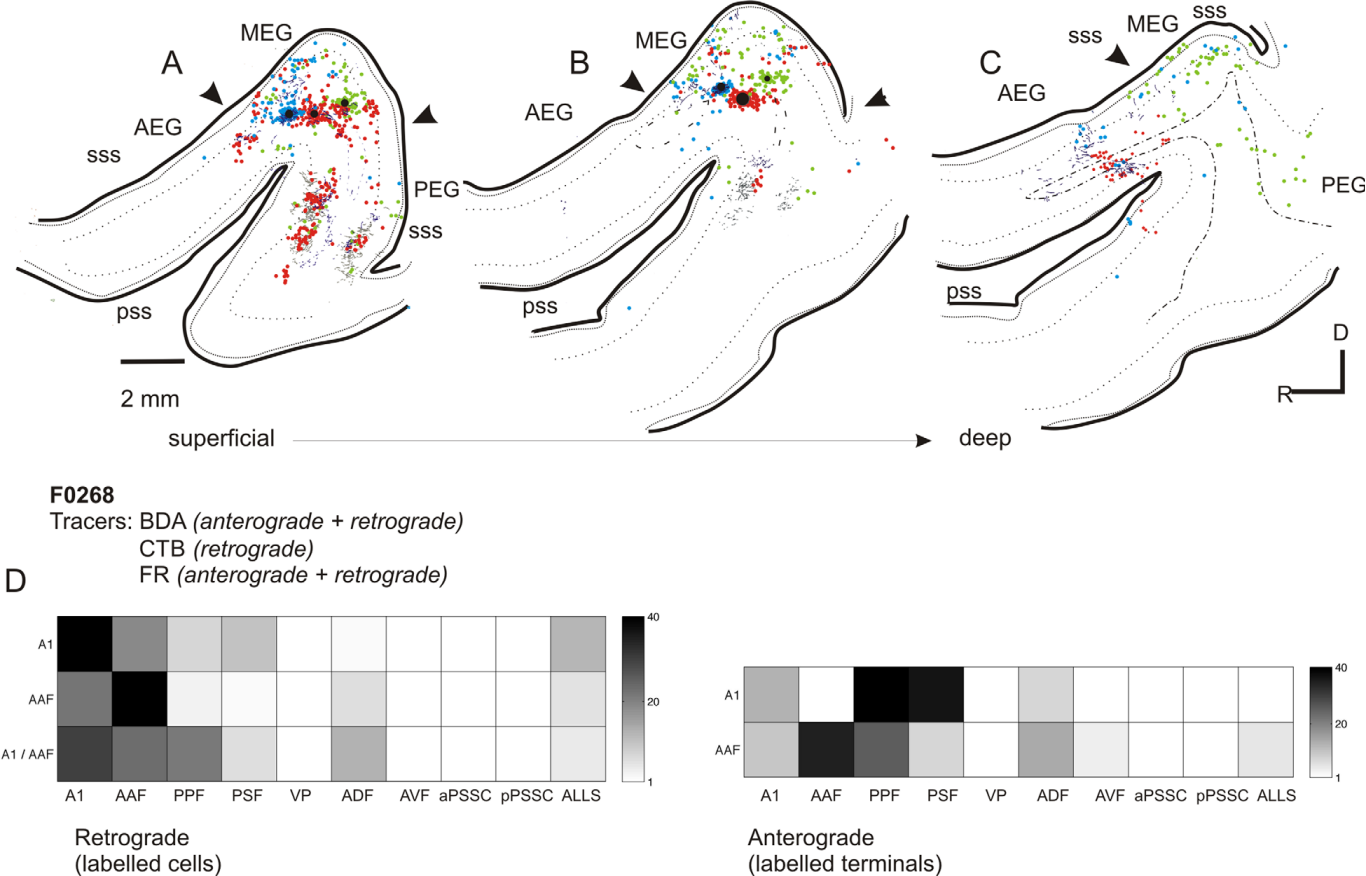
Summary of ipsilateral labeling for case F0268. **A–C:** Labeling from all three tracers used in Figure 3 (F0268, frequency‐matched injections into MEG) overlaid onto sections at three different depths (A, most superficial; C, deepest). **D:** Summary of the pattern of anterograde and retrograde labeling for this case. For abbreviations, see list. Scale bar = 2 mm in A (applies to A–C).

The topography of these connections is more clearly demonstrated in the case illustrated in Figures 3 and 4, where the cortex has been flattened and cut in the tangential plane. Here, three frequency‐matched injections (CF = 7 kHz) were placed at different rostrocaudal positions within the MEG. Figure 3B–M illustrates the pattern of labeling that resulted from each of the injections, with the sections ordered from the most superficial to the deepest, and the labeling associated with each tracer represented in a separate column. The left column shows the anterograde and retrograde labeling (dark and light green, respectively) from an FR injection into the A1, the central column is the retrograde labeling (red) resulting from an injection of CTB into the center of the frequency‐matched region, and the right column depicts the anterograde and retrograde labeling (dark/light blue) following an injection of BDA into the AAF. In Figure 4A–C, the labeling from all three tracers is overlaid. This highlights a band of interdigitating label, corresponding to a putative isofrequency lamina, which runs across the three injection sites. Scattered labeling is found in the MEG away from this band, and in the ALLS (Fig. 3F–H,K,M).

Tracer deposits within the MEG always produced labeling on the PEG consistent with a strong projection from primary to posterior fields. On the PEG, clearly defined anterograde labeling was observed following injections of BDA and FR (CTB is only transported retrogradely), with both injections producing clearly overlapping terminal fields (Fig. 4A–C). Consistent with the physiological descriptions of the organization of the PPF and PSF, two discrete patches of overlapping terminals were separated rostrocaudally by a terminal‐free zone. These separated terminal fields can also be observed in the coronal sections in Figure 2 (most clearly in the composite schematic shown in Fig. 2B). Because we did not use different fluorescent markers to label the three tracers in the same section, we were not able to visualize double‐ or triple‐labeled terminal fields. Consequently, whether these broadly overlapping and frequently intermingled (see Fig. 11I for an example) terminations represent convergent projections or are interdigitated in the same area remains to be elucidated. Little or no labeling was found at the most ventral extreme of the PEG, corresponding to field VP. Comparison of frequency‐matched injection sites in the A1 and AAF showed that injections in the A1 resulted in a heavier projection to the PPF and PSF than did those in the AAF (Fig. 4D).

The pattern of labeling in the AEG after injections in the MEG was very similar irrespective of whether the injections were placed in the A1 or AAF (compare labeling in the MEG in Fig. 3H, F, and D and in the AEG in Fig. 3B, C, and D), although a quantitatively smaller projection was observed from the A1 compared with the AAF. The label was scattered and interdigitating rather than restricted and overlapping as in the PEG.

Quantification of the projections resulting from injections into the MEG showed that the strongest projections from the A1 and AAF were within the auditory core, both within the injected field and between the A1 and AAF. In addition, strong reciprocal connections were observed with the PPF and PSF. The finding that frequency‐matched injections in the primary fields produce discrete, overlapping areas of labeling on the posterior gyrus suggests that connections exist between frequency‐matched areas of these tonotopically organized auditory cortical fields. Injections in the high‐frequency MEG (Fig. 2) produced patches of labeling in more rostral and caudal aspects of the PEG than lower frequency injections (Figs. 2, 3), after which labeling was located closer to the center of the PEG, again in keeping with the known tonotopic organization. Smaller projections were observed from the primary fields to the ADF, and connections between the primary fields and the AVF, VP, and PSSC were virtually absent. Injections in the A1 tended to produce heavier labeling in the posterior fields than those in the AAF, whereas AAF injections produced heavier labeling in the ADF than those made in the A1 (Fig. 4). Thus, despite the partial overlap in the labeling patterns, these data provide evidence for the existence of parallel projections originating from the A1 and AAF.

### Injections in MEG: callosal connectivity

**Figure 5:**
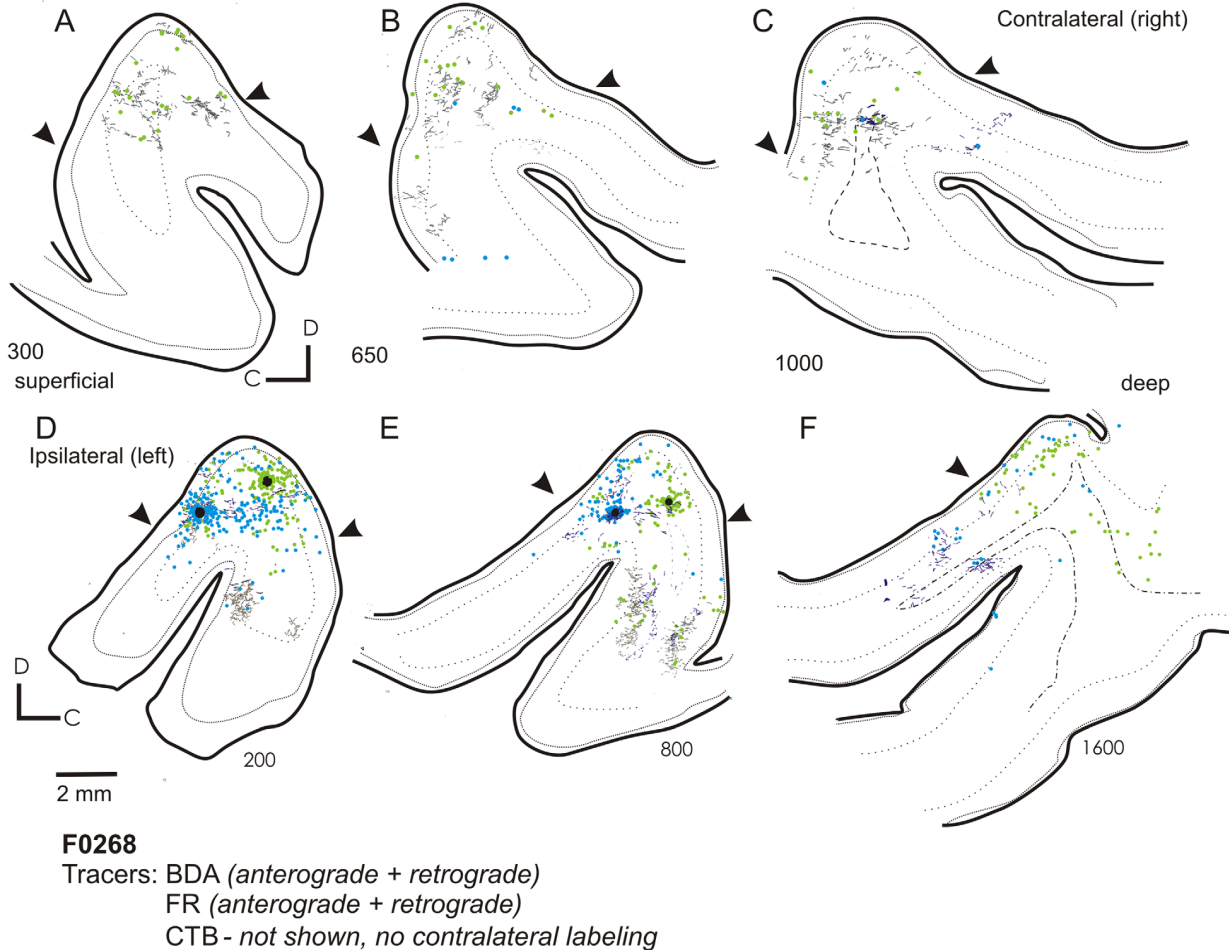
Labeling in the contralateral auditory cortex following injections into a 7‐kHz isofrequency lamina in the MEG. Drawings show the contralateral labeling after the frequency‐matched injections shown in Figures 3 and 4. **A–C:** Labeling from the FR injection and BDA injections (A–C, superficial to deep) is shown with retrogradely filled cells plotted as light green and blue circles, respectively; the dark green and blue lines represent the corresponding terminal fields. **D–F:** Ipsilateral labeling from similar depths is shown for comparison. The CTB injection did not produce any contralateral label. For abbreviations, see list. Scale bar = 2 mm in D (applies to A–F).

Previous studies in ferrets documenting callosal projections from the MEG revealed multiple bands of anterograde label running orthogonal to the main tonotopic axis (Wallace and Bajwa, 1991; Pallas and Sur, 1993; Wallace and Harper, 1997). Figure 5 shows the contralateral labeling found following the injections illustrated in Figures 3 and 4. The FR injection produced the most comprehensive anterograde transport, and multiple orthogonal bands of label could be observed contralateral to the injection site (Fig. 5A,B), consistent with these previous studies. Additionally, anterograde label was evident on the posterior bank, mirroring that observed ipsilaterally.

The pattern of retrograde contralateral labeling resulting from tracer injection in the MEG generally mirrored that observed in the ipsilateral auditory cortex, although there were many fewer labeled cells. As in the ipsilateral MEG, there was a band of retrograde and anterograde labeling that ran rostrocaudally through the MEG, with further labeling found in the sss at the dorsal extreme of the gyrus (Fig. 5A,B) and in the PSF at a location corresponding to that observed in the ipsilateral auditory cortex. Again, as on the ipsilateral side, the BDA injection in the AAF resulted in scattered labeling in the contralateral ADF, but neither the BDA nor the FR injection produced any labeling in the contralateral PPF.

### Injections in the PEG: PPF

**Figure 6:**
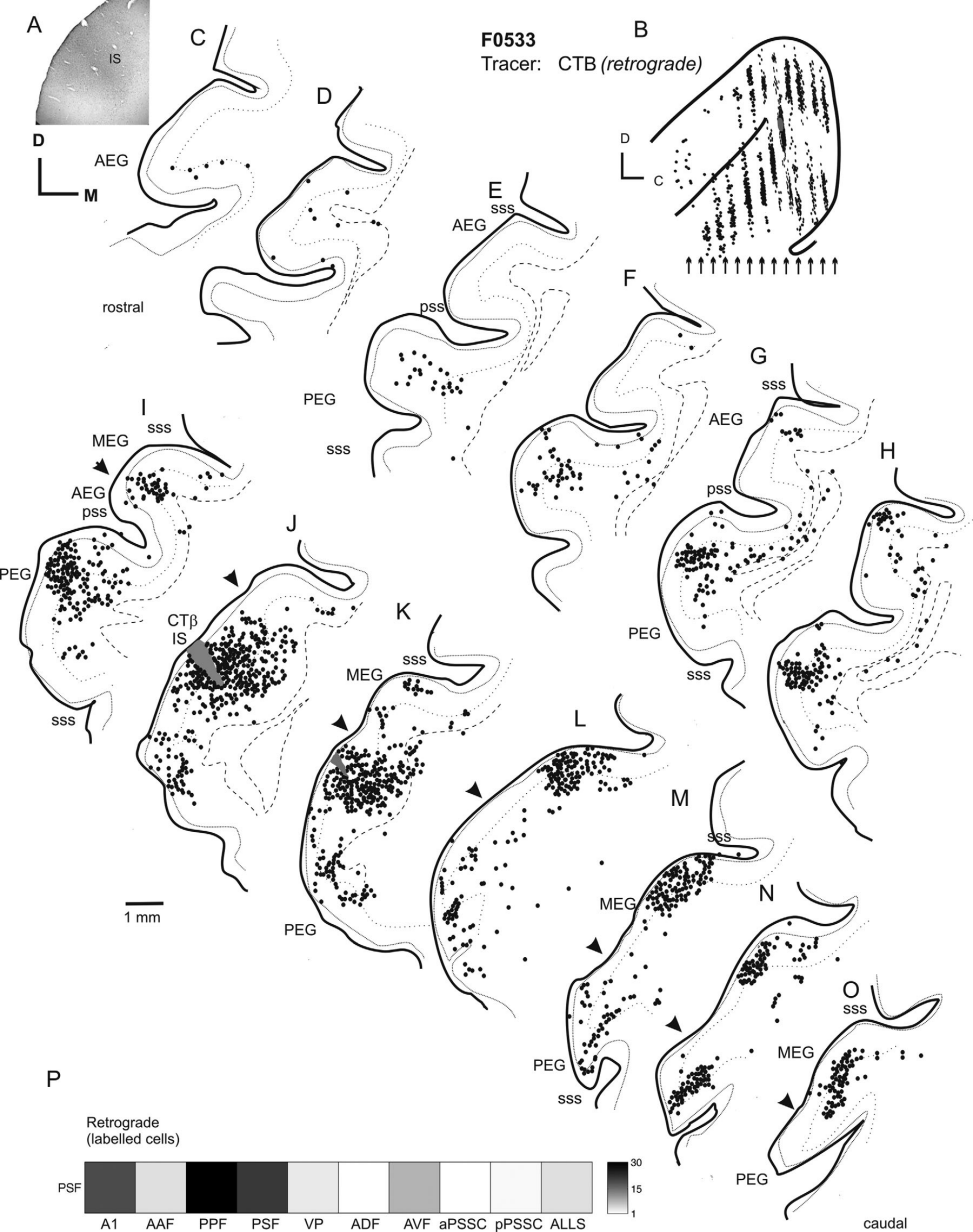
Ipsilateral labeling resulting from an injection of CTB into the posterior pseudosylvian field (PPF). **A:** Photograph showing the location of a large injection of CTB into PPF. **B:** Schematic showing the injection site, resulting labelling, and location of the coronal sections (arrows) drawn in C–O. **C–O:** Coronal sections from ipsilateral cortex showing the distribution of retrograde labeling. Black circles indicate the location of back‐filled cells, and the injection site is marked in gray. **P:** Quantification of retrograde labeling. For abbreviations, see list. Scale bar = 1 mm in J (applies to A–O).

Although previous studies have demonstrated projections from the MEG to the PEG (Pallas and Sur, 1993), none have placed tracer injections into physiologically identified PEG regions. We therefore targeted our injections to the PPF and PSF. Figure 6 illustrates the pattern of labeling observed following a large CTB tracer injection in the PEG. Recordings made near this injection site had CFs of ∼8 kHz, with more rostral recording locations having higher CFs. This injection was therefore centered within the PPF. As can be seen from Figure 6, this large injection site produced retrograde labeling across much of the dorsal MEG (Fig. 6J–N), as well as in the PEG, including both the PSF and VP (Fig. 6I–M,N). Back‐filled cells were also observed within the ADF and to a lesser extent in the AVF (Fig. 6B–F). Labeling was also present in the ALLS and pPSSC, but was largely absent from the aPSSC. Finally, in keeping with the CF of the injection site, virtually no labeling was present in the ventral MEG (where the low‐frequency A1 and AAF are located; Fig. 6L–M) or in the central PEG, where a low‐frequency area separates the PSF and PPF (Fig. 6J–L), In this case, no anterograde labeling was observed due to the retrograde nature of the tracer used.

**Figure 7:**
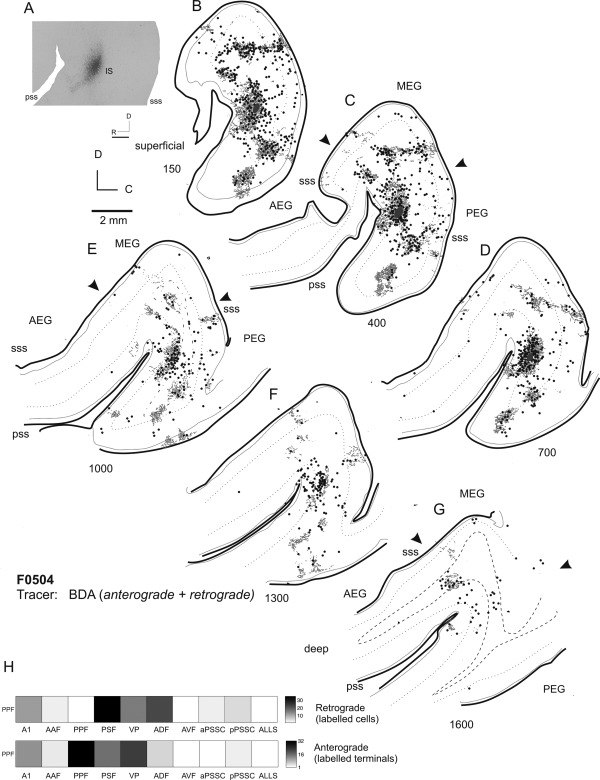
Flattened tangential sections of ipsilateral cortex illustrating the labeling resulting from an injection of BDA into the PPF. **A:** Photograph showing injection site location. **B–G:** Sections ordered from most superficial to deepest in 300‐µm intervals. Back‐filled cells are plotted as black circles, and terminal fields are shown in gray. **H:** Quantification of anterograde and retrograde labeling. For abbreviations, see list. Scale bar = 1 mm in A; 2 mm in E (applies to B–G).

Figure 7 illustrates the labeling pattern after a smaller injection of BDA into the PPF, this time in flattened cortex. This injection resulted in a band of labeled cells running approximately two‐thirds of the way across the MEG from the caudal edge of the gyrus (Fig. 7B,C). This discrete band of labeling likely represents an isofrequency lamina within the A1. The labeling observed after PPF injections confirms the reciprocal connectivity with the primary auditory cortical fields that was suggested by the MEG injections (compare Figures 4 and 7), and illustrates the stronger connection that arises from the A1 than from the AAF.

The PPF is also reciprocally connected with the VP, as indicated by the retrograde and anterograde labeling observed in this field (Fig. 7D–F). Some labeling was also present in the AEG, in the anterior part of the ADF, but was absent in the AVF. Sparse labeling was also found in the pPSSC, but was almost absent in the aPSSC (e.g., Fig. 7D).

### Injections in PEG: PSF

**Figure 8:**
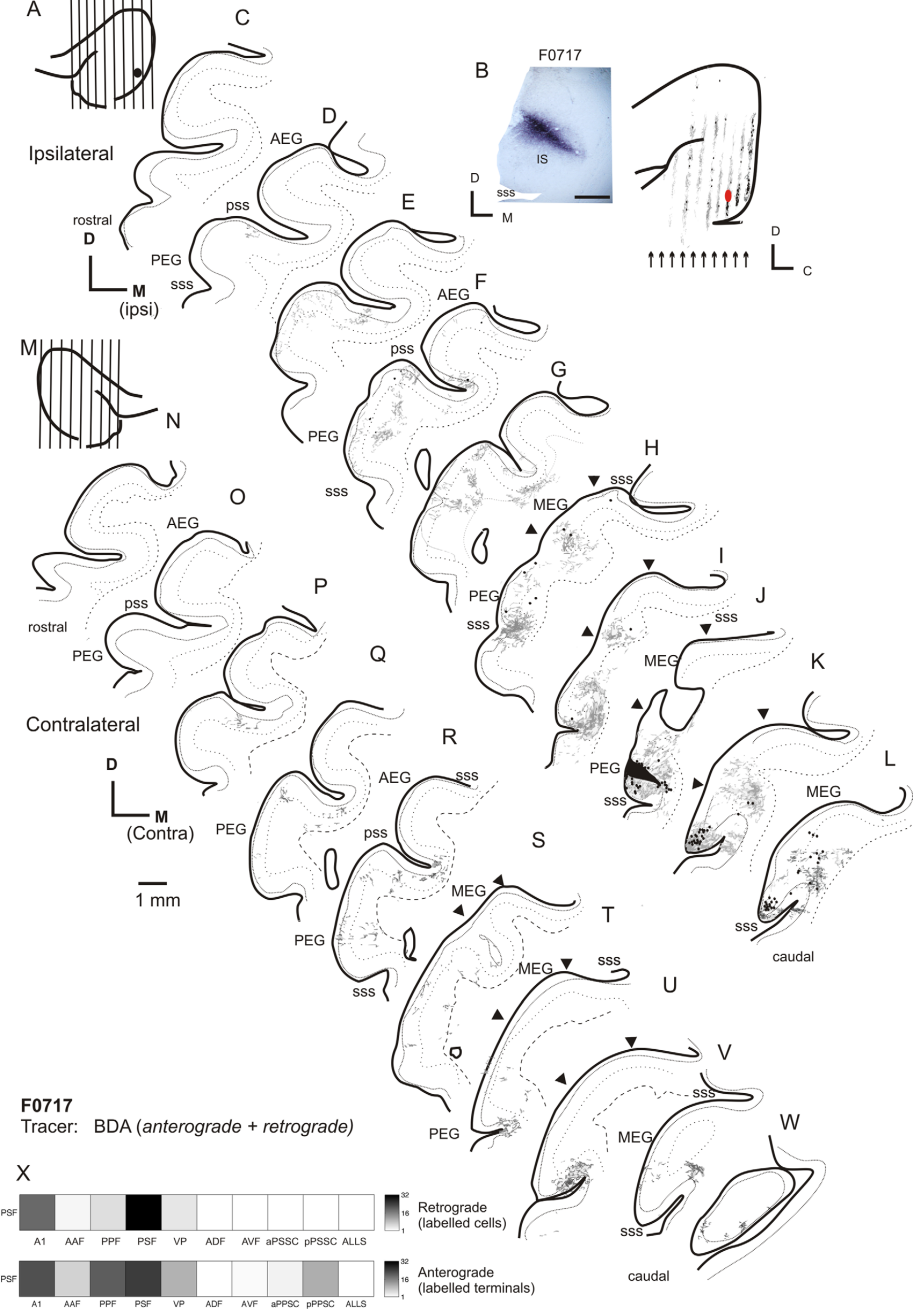
Ipsilateral and contralateral labeling after an injection of BDA into the PSF. **A,B:** Schematic and photograph of the injection site. B: Location of the coronal sections shown in C–L, summarizing the topology of the resulting labeling. **C–L:** Drawings of coronal sections in ipsilateral cortex ordered from rostral to caudal. Back‐filled cells are shown in black, and terminal fields are shown in gray. **M:** Schematic showing the location of the illustrated coronal sections (**N–W**) in the contralateral cortex of the same animal. **X:** Quantification of the labeling in the ipsilateral cortex. For abbreviations, see list. Scale bar = 1 mm in B; 1 mm in Q (applies to C–W).

The pattern of labeling resulting from injections in the PPF suggests that the connectivity of this region is similar to that of the primary fields (notably the A1), with strong projections to the A1 and field PSF. Next, we placed injections of neural tracer into the PSF, to compare the connectivity patterns of the posterior fields. Figure 8 illustrates the labeling in both ipsilateral and contralateral auditory cortex resulting from a small injection of BDA in the PSF. A pattern of labeling was found similar to that observed following injection of tracer into the PPF. Once again, labeled terminal fields were evident in the caudal half of the MEG (seen as labeling at the same dorsoventral location within the MEG in Fig. 8H,I,K). There were terminal fields running along the whole of the ventral extreme of the PEG, where the field VP is located (Fig. 8F,H,I), which were found predominantly in the superficial layers (II/III). Relative to the PPF, tracer injections in the PSF produced heavier labeling in both the aPSSC and the pPSSC (compare Fig. 7E,F with Fig. 8E–G).

### PEG injections: callosal connectivity

Again, the pattern of labeling produced in the contralateral cortex generally mirrored the pattern of the strongest labeling seen in the ipsilateral cortex. Figure 8M–W shows an example of the labeling observed after an injection of BDA into the PSF. Although the pattern of contralateral labeling mirrored that seen ipsilaterally in most sections, there was an exception to this within the primary fields, which tended to exhibit much less labeling than in the ipsilateral cortex; compare sections in Figure 8S, T, and U, in which MEG label is almost entirely absent, with Figure 8G, H, and J, in which there are clear terminal fields in the MEG. PPF injections (data not shown) produced a similar pattern of contralateral terminal labeling, except that a clear band of labeling was found in the contralateral A1, suggestive of connections between isofrequency laminae on each side.

### Injections in AEG: ADF

**Figure 9:**
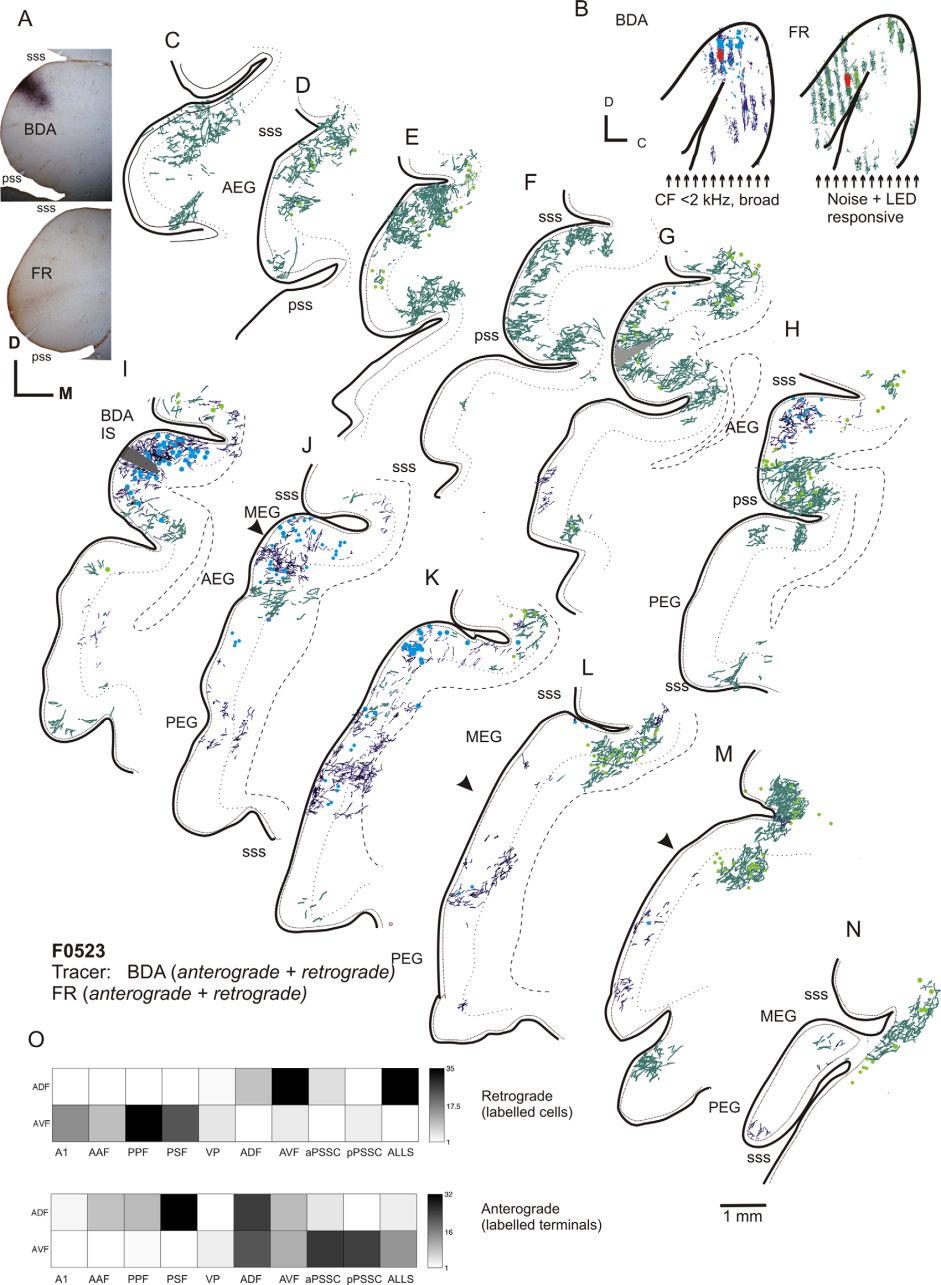
Ipsilateral labeling after injections into the anterior dorsal and ventral fields. **A:** Photograph showing the location of the injection sites. **B:** Schematic showing injection site locations (red circles) and distribution of labeling for injections of BDA (blue label) into the ADF and FR (green label) into the AVF. Neither of these injection sites encroached on the pseudosylvian sulcus. Arrows indicate the locations of alternate coronal sections plotted in C–N. **C–N:** Drawings of coronal sections showing labeling in auditory cortex; terminal fields are plotted as dark blue lines (BDA) or dark green lines (FR), with retrograde labeling shown as light blue circles (BDA) or green circles (FR). **O:** Quantification of the labeling. For abbreviations, see list. Scale bar = 1 mm in N (applies to A–N).

To determine whether the projection patterns between areas on the anterior bank and those on the posterior bank differed from one another, in six instances we placed injections into the AEG. Figure 9 shows the labeling resulting from an injection of BDA into the ADF and one of FR into the AVF. The injection of BDA into the ADF labeled cells and terminals within the anterior MEG and in the dorsal sss (Fig. 9I,J). In contrast, the injection made at the ventral extreme of the AEG did not back‐fill cells within the MEG other than in the banks of the sss where the ALLS is located (Fig. 9K–M).

**Figure 10:**
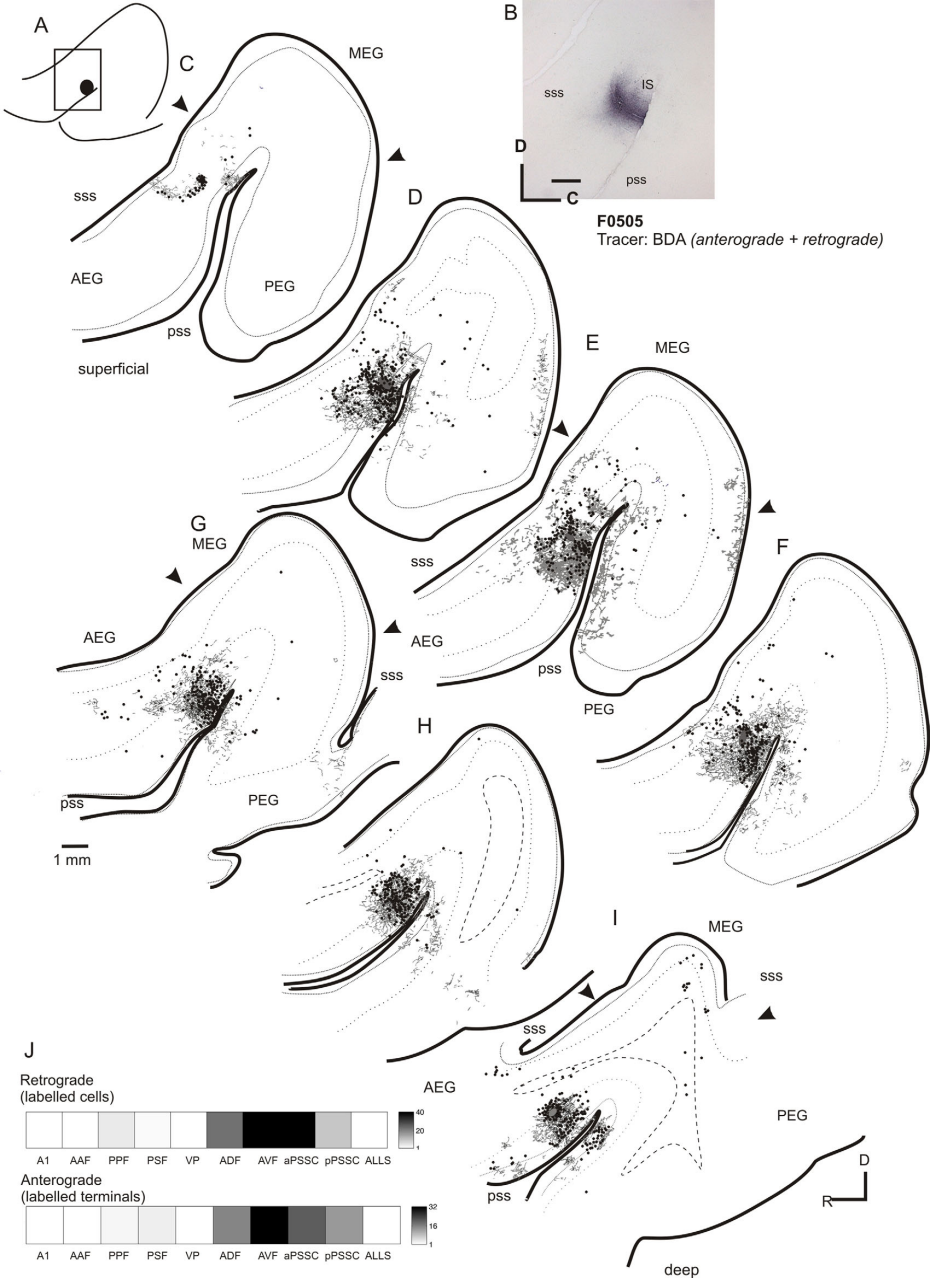
Flattened tangential sections showing the distribution of labeling after an injection of BDA close to the border of the ADF and aPSSC. **A,B:** Location of the injection site (A) and photograph of the injection (B). **C–I:** Drawings showing the labeling in auditory cortex organized from the most superficial to the deepest. Back‐filled cells are shown in black, and terminal fields in gray. **J:** Quantification of the labeling. For abbreviations, see list. Scale bar = 1 mm in B; 1 mm in G (applies to C–I).

Projections from the ADF to the PSF were more numerous (Fig. 9K–M) than those from the ADF to the PPF, which were relatively sparse (Fig. 10F–I; see also Figs. 7 and 8). The ADF and PSF both projected to the PSSC, but the ADF projected exclusively to the aPSSC (Fig. 9I; see also Fig. 10D–I), whereas the PSF projected to both the aPPSC and pPSSC (Fig. 8D–G), highlighting the fact that the anterior and posterior banks have distinct patterns of connectivity. Injections in the ADF also revealed that this area was weakly connected with the VP (Fig. 9L).

**Figure 11:**
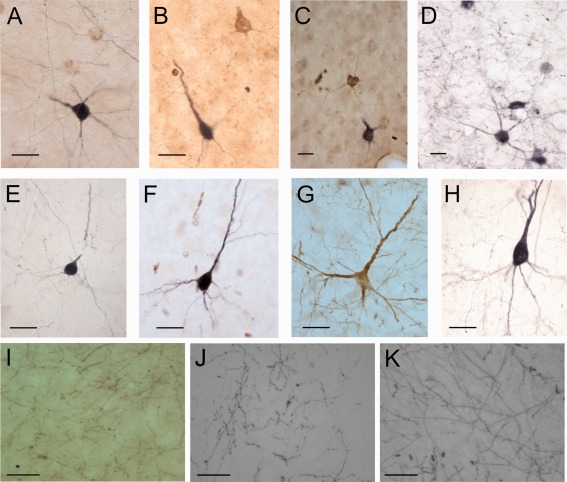
Examples of labeled neurons. **A:** F0268, FR (brown) and BDA (black) injection in A1 and AAF, labeled cells in A1. **B:** F0532, FR (brown) and BDA (black) injection in A1 and AAF; cells in the AAF. **C:** F0532, FR (brown) and BDA (black) labeled cells in the PPF. **D:** F0504 BDA injection in the PPF; labeled cells and terminals in putative isofrequency laminae in A1. **E:** F0505, BDA injection in the AEG; labeled cell in the PPF. **F:** F0523, BDA injection in the ADF; labeled cell in the ALLS. G: F0523, FR injection in the AVF; labeled cell in the ALLS. **H:** F0523, BDA injection in the ADF; labeled cell in the aPSSC. **I:** F0268, terminal fields in the PPF after injections of FR (brown) in A1 and BDA (black) in the AAF. **J:** F0717, BDA injection in the PSF; terminal field in the pPSSC. **K:** F0523, FR injection in the AVF; terminal field in the aPSSC. For abbreviations, see list. Scale bar = 50 µm in A–H; 25 µm in I–K.

### Injections in AEG: AVF

The AVF also projects to the VP (Fig. 9G–I) but does not innervate or receive input from the PPF or PSF. AVF tracer injections produced dense terminal labeling caudal and dorsal to the suprasylvian sulcus in area SSY (Fig. 9L,M). Injections placed in the ADF and AVF produced a region of overlapping labeling between the injection sites, which encompassed both the gyrus itself and the aPSSC (Fig. 9G–I).

Figure 10 illustrates the results of a tracer injection into the AEG, centered in the AVF, but that included the aPSSC and encroached on the ADF. In contrast to the ADF injection in Figure 9, there was scarcely any labeling in the MEG, and what little was present was restricted to the ALLS (Fig. 10I). Injections placed in the ADF and aPSSC revealed heavy projections to and from the immediately neighboring cortex within the pss and on the caudal half of the AEG (Fig. 10). Terminal fields were evident on the PEG, most notably in the sulcus caudal to the PSF (Figs. 9L–M, 10D,E). Because terminal fields were not observed in the PSF following injection of tracer into the AVF, it seems likely that the projection to the PSF arises from the aPSSC.

### AEG injections: callosal connectivity

Labeling in the contralateral cortex mirrored that observed in the ipsilateral cortex. Patterns of labeling from injections in both the ADF and AVF were very symmetrical between the cortices, with retrogradely cells and terminal fields located in corresponding locations in each cortex (not shown).

### Anterolateral suprasylvian sulcus

The ALLS has previously been identified on the basis of its cytoarchitecture (Manger et al., 2008; Homman‐Ludiye et al., 2010), but neither physiological nor connectional studies have been performed within this area. We found that the ALLS, which lies dorsal to the A1 and AAF, within the lateral bank of the sss, is reciprocally connected with both primary auditory areas (Fig. 4B–D) and the PPF (Fig. 6H–J), and to a lesser extent with the PSF (Fig. 8F,H) and ADF (Figs. 9I–K, 11G).

### Laminar distribution of projections

Projections from the A1 and AAF were reciprocal and terminated predominantly, but not exclusively in layers II/III. Projections from both the A1 and AAF to the posterior fields PPF and PSF targeted both supragranular and infragranular layers (Figs. 2H–J, 3G,H). In contrast, projections from the A1 to ADF terminated mostly in layers II and III (Fig. 3B), whereas those from the AAF terminated in both upper and lower layers (e.g., Fig. 3J,M).

Projections from the PPF also tended to target upper and lower cortical layers. This was the case for projections from the PPF to the primary fields A1 and AAF (Fig. 7B–D), PSF (Fig. 7B,E) and the pPSSC. Projections from the PSF to the A1 targeted all cortical depths (Fig. 8H,I), as did those to the pPSSC. In contrast, projections to the PPF and ADF (Fig. 8D–F) were predominantly to the upper (II/III) layers.

Projections from the ADF to the AVF and aPSSC targeted mostly layers II and III (Fig. 9I), as did those from the ADF to the AAF (Fig. 9J,K) and PPF (Fig. 9H,I). Projections from the ADF to the PSF spanned the cortical layers (Fig. 9K,L). Finally, AVF neurons terminated at all depths in the aPSSC and in layers II and III of the pPSSC and VP (Fig. 9H,I).

**Figure 12:**
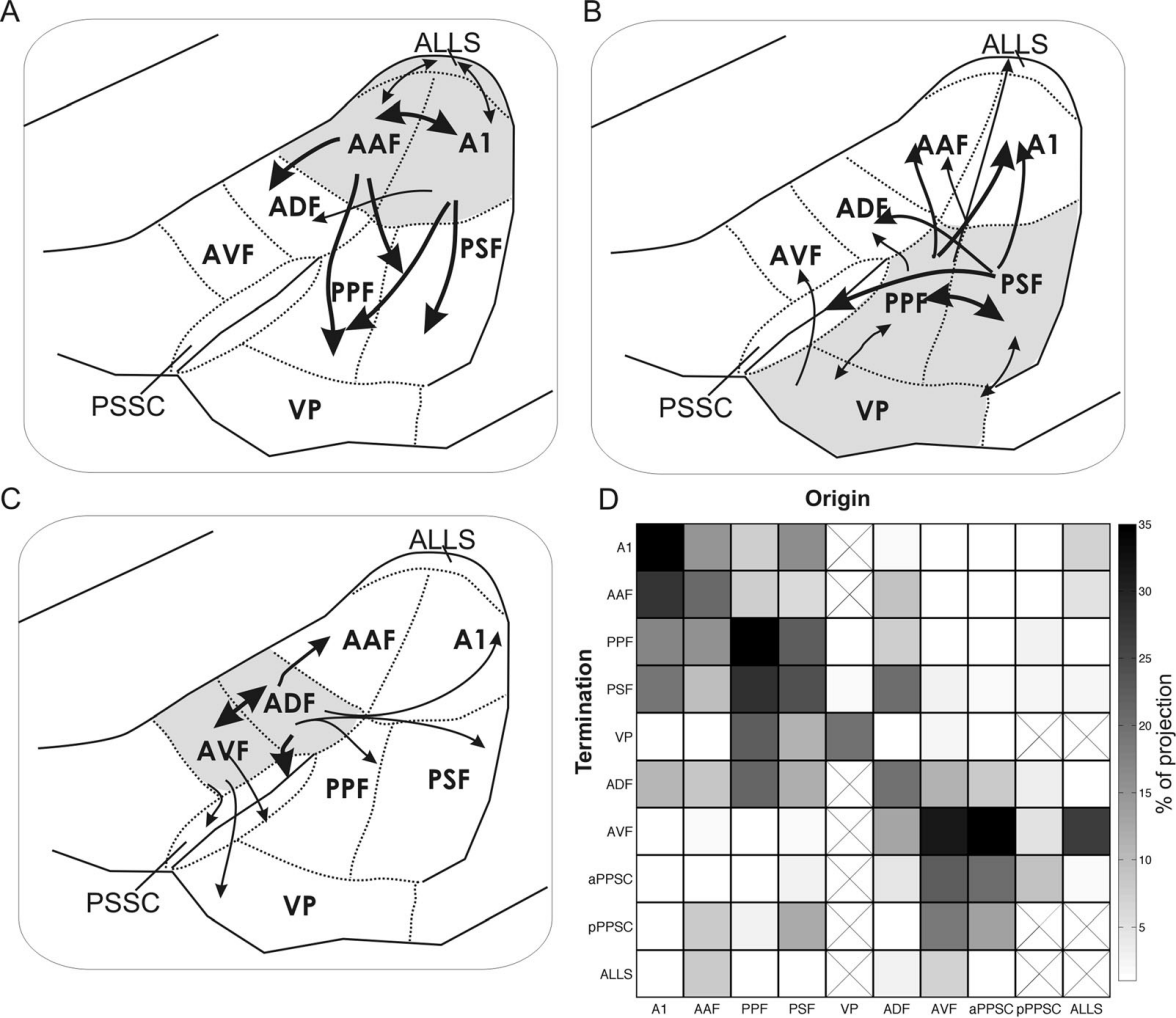
Summary of connections between auditory cortical fields. **A:** Projections originating from A1 and AAF located in the MEG. The relative strength of the connection is indicated by the width of the arrow. **B**: Projections within the fields on the PEG (PPF, PSF, and VP), and from these fields to areas on the MEG and AEG. **C:** Projections within the fields on the AEG (ADF and AVF), and from these fields to areas on the MEG and PEG. **D:** Summary of all projections observed. Boxes marked with an X indicate possible connections that we were unable to observe as we did not target injection sites at these areas. For abbreviations, see list.

Figure 11 illustrates the morphology of typical labeled cells and terminal fields throughout the auditory cortex. Figure 12 summarizes the projection patterns observed across all experiments. These are represented in two ways: Figure 12A, B, and C, respectively, illustrates the projections identified within and beyond the MEG, PEG, and AEG. Figure 12D summarizes the relative strengths of the connections between each of the cortical areas and is based on the summary panels shown for each of the individual animals.

## DISCUSSION

We placed deposits of anterograde and retrograde neural tracers into the six physiologically identified areas, to determine the projection patterns between these fields and others on the EG. Previous tracer studies in ferret revealed that these areas are innervated by the ventral division of the MGB (Pallas et al., 1990), and that multiple areas, predominantly on the PEG, but also on the AEG, receive connections from the MEG (Wallace and Bajwa, 1991; Pallas and Sur, 1993; Gao and Pallas, 1999). Since these studies were completed, we have gained a deeper understanding of the functional organization of the auditory cortex as assessed by responses to both simple (Kowalski et al., 1995; Nelken et al., 2004; Bizley et al., 2005, 2007a) and complex stimuli (Nelken et al., 2008; Bizley et al., 2009, 2013; Atiani et al., 2014), warranting a more comprehensive investigation of the connectivity within the auditory cortex. Our anatomical investigations support the idea that distinct anterior and posterior processing pathways exist and extend our understanding about the organization of the auditory cortex in the ferret, thus providing crucial information to facilitate cross‐species comparisons. Our findings are summarized in Figure 12, which demonstrates the key projection pathways from each of the fields investigated. This illustrates that the strongest connections are between fields located within the same, cytoarchitectonically distinct, region of the EG. Furthermore, the primary areas have dense terminal fields in the adjacent nonprimary areas (AAF to ADF and A1 to PPF and PSF; Fig. 12A), whereas the secondary areas make more widespread connections (Fig. 12D).

### Tonotopic organization

One of the key objectives of this study was to determine whether the patterns of anatomical connectivity were consistent with the tonotopic organization described physiologically (Kelly et al., 1986; Nelken et al., 2004; Bizley et al., 2005). Optical imaging and single‐unit recordings demonstrate that two tonotopic fields, the A1 and AAF, are located within the MEG with neurons representing high sound frequencies present at the apex of the gyrus and neurons tuned to lower sound frequencies situated more ventrally (Bizley et al., 2005; present results, Figs. 2, 3, 4, 5, 6). On the PEG there are two tonotopically organized fields, the PPF and PSF, whose frequency gradients reverse across a low‐frequency–preferring area that runs dorsoventrally along the middle of the gyrus. The frequency‐response areas constructed from the responses of neurons recorded in the PPF and PSF are often as narrow as those observed in the primary fields, although a higher incidence of more complex tuning exists, such as nonmonotonic rate‐level tuning at the CF of the neuron (Bizley et al., 2005).

The anatomical data presented here demonstrate that the core auditory fields and the two posterior bank areas PSF and PPF are reciprocally connected. Moreover, the A1, AAF, PPF, and PSF are innervated by the ventral division of the MGB, with the posterior fields additionally receiving input from the dorsal and medial subdivisions of the MGB (Nodal, Bajo, Bizley, and King, unpublished observation; Bizley, 2005). Injections into the PPF produced stronger labeling in A1 than injections into the PSF, whereas those in the PSF produced stronger labeling in the PSSC. The anatomical data presented here also support the parcellation of the anterior bank into at least three areas: the ADF, which is not tonotopically organized and receives inputs predominantly from the AAF, PSF, and AVF; the AVF, a multisensory area whose inputs from the EG arise principally from the ADF; and the aPSSC, which is innervated by the PSF, ADF, and AVF. Each of these regions also receives cortical inputs originating outside the EG (Ramsay and Meredith, 2004; Manger et al., 2005; Bizley et al., 2007a).

Our anatomical data also confirm the existence of a third posterior bank field, the VP, which is located at the ventral extreme of the posterior gyrus (Bajo et al., 2007). The VP differs from the PPF and PSF in that it does not receive inputs from, or project to, the primary areas located in the MEG, likely placing it further up the cortical processing hierarchy than the other fields on the posterior bank. Finally, our data suggest that the field located in the lateral wall of the medial suprasylvian sulcus (the ALLS; Manger et al., 2008), which can be cytoarchitectonically differentiated from the fields on the gyrus (Homman‐Ludiye et al., 2010), is likely to be sound responsive.

### Distinguishing the A1 and AAF

The ferret, unlike most mammalian species, does not display a clear tonotopic reversal between the A1 and AAF. There is considerable individual variability in the organization of the A1 and AAF, with about 20% of cases showing a reversal between the fields and the remainder of animals showing parallel gradients (Nelken et al., 2004; Bizley et al., 2005). Although there are differences in the response latencies of neurons within these two fields (Kowalski et al., 1995; Bizley et al., 2005), the lack of a consistent tonotopic reversal does raise the question of whether the AAF should be regarded as a separate field. When frequency‐matched injections were made into the MEG of the same animals, the resulting patterns of labeling demonstrate a tendency for the AAF to connect more strongly to the ADF and the A1 to the posterior fields. This distinct pattern of connectivity to other parts of the EG is consistent with the idea that the A1 and AAF comprise independent connectional systems, as proposed for cat auditory cortex (Lee et al., 2004), in which studies using cortical cooling have provided evidence for functional differences between the A1 and AAF (Malhotra and Lomber, 2007; Lomber and Malhotra, 2008). Detailed investigation of the cortico‐thalamic connectivity (ideally combined with gene expression studies such as in Storace et al., 2010) will provide further information about the potential differences in these processing streams.

### PSSC

It has previously been demonstrated that the PSSC is innervated by both the somatosensory and the visual cortex (Ramsay and Meredith, 2004; Manger et al., 2005; Bizley et al., 2007b). The data presented here show that this area is also innervated by acoustically responsive areas on the EG, particularly the ADF and AVF on the AEG and the PSF on the PEG. The anterior bank of the PSSC projects to the superior colliculus (Bajo et al., 2010a), and is likely to be homologous to the anterior ectosylvian sulcal field (fAES) of the cat (Jiang et al., 1996; Manger et al., 2005). In accordance with the suggestion that cat fAES may be specialized for spatial processing (Middlebrooks et al., 1998; Malhotra and Lomber, 2007; Las et al., 2008), it has been shown that inputs to the aPSSC from the visual cortex arise predominantly from area SSY (Bizley et al., 2007a), which is thought to be specialized for visual motion processing (Philipp et al., 2006). Previous studies of the PSSC have not demarcated the anterior and posterior banks. Nevertheless, the data presented in Ramsay and Meredith (2004) show that whereas projections from the somatosensory cortex target both banks, those from the visual cortex target only the anterior bank. The patterns of anatomical connectivity between the auditory cortical fields are consistent with the idea that the two banks of the sulcus should be considered as anatomically distinct: the anterior bank is predominantly connected with the anterior fields ADF and AVF, whereas the PSF connects predominantly with the pPSSC.

### ALLS

Tracer injections in the primary auditory cortical fields and in both the PPF and the PSF resulted in labeling patterns consistent with the existence of isofrequency laminae within these regions, together with additional scattered labeling at non‐homotopic sites. However, a consistent finding was that there was also considerable labeling spanning the sss around the MEG where the ALLS is located. This labeling was often evenly distributed around the whole dorsal tip of the sss, extending ventrally to roughly the border between the MEG and the secondary fields. Labeling was also observed in these areas after tracer injections in the ADF and aPSSC. Therefore the ALLS is likely to be an additional nonprimary, acoustically responsive area, but confirmation of this hypothesis requires physiological investigation.

### Processing networks in auditory cortex

Auditory information appears to be processed in series and in parallel throughout the cortical fields examined. Auditory information enters the auditory cortex in parallel via multiple auditory areas including, but not restricted to, the A1 and AAF. Auditory activity is propagated through two separate but overlapping pathways. In one case, information enters the auditory cortex via the AAF and from there passes through an anterior pathway from the AAF to the ADF and AVF. These anterior areas are characterized by short response latencies and broad frequency tuning (Bizley et al., 2005) and may be well suited to temporal processing tasks. Lemniscal input is additionally supplied from the MBGv to the A1, and this information is then relayed from the A1 to the PPF and PSF and from them to the VP. The tonotopically organized PPF and PSF also receive direct input from the MGBv (Bizley, 2005). Neurons located in the posterior fields have longer latencies than those in the AEG and frequently display a rich variety of temporal profiles (Bizley et al., 2005). In addition to input from the MGBv, these areas are innervated by the nonlemniscal auditory thalamus (Nodal, Bizley, Bajo, and King, unpublished observations). The A1 and AAF are reciprocally, although asymmetrically, connected, and interactions between these processing pathways occur at all stages. In particular, both the anterior and posterior banks of the pseudosylvian sulcus are innervated by both the PSF and the AVF.

We have previously demonstrated that within the different regions of ferret auditory cortex, spatial sensitivity to auditory, visual, and bisensory stimulation is greatest in the ADF (Bizley and King, 2008), whereas PSF neurons were the most likely to have their auditory spatial tuning enhanced by the addition of a spatially and temporally congruent visual stimulus (Bizley and King, 2008). The anatomical data presented here suggest that the PSF is ideally positioned to integrate spatial information conveyed by neurons in fields on the AEG. Injections that included the aPSSC produced terminal fields that ran along the caudal end of the PEG, where the field PLLS is proposed to lie (Manger et al., 2004), and that received inputs from the PSF. Indeed, our data suggest that neurons within the bank of the pseudosylvian sulcus may act as a gateway that facilitates integration of information between neurons located in the anterior and posterior auditory cortical processing pathways.

The multiple inputs to the auditory cortex, and the multiple pathways through the auditory cortex, are possible reasons why the immediate and temporary nature of cortical inactivation via cooling produces such pronounced behavioral deficits when compared with the rather more nuanced changes observed after more prolonged forms of inactivation or permanent lesions (Heffner, 1997; Smith et al., 2004; Bizley et al., 2007b; Malhotra and Lomber, 2007; Nodal et al., 2010, 2012). These multiple pathways potentially provide the auditory cortex with a basis by which considerable compensatory plasticity can occur, with information organized in a frequency‐specific way still gaining access to the auditory cortex even in the absence of an intact auditory core. In addition, the multiple points at which these parallel pathways interconnect provide a further opportunity for information to be rerouted through the auditory cortex.

### Homologies with other species

The A1 is highly conserved across mammals, and, whereas there are species‐specific differences in the orientation of the frequency axis, this area is by definition tonotopically organized and innervated by neurons in the MGBv (reviewed in Lee and Winer, 2011). However, most species, including ferrets, have multiple tonotopically organized auditory cortical fields, more than one of which receives direct MGBv input. Identifying homologous, or at least analogous, fields between species is essential, to generalize physiological or behavioral findings made in any one study.

Because they are both carnivores, it might be expected that ferret auditory cortex would most closely resemble that of the cat. It has been suggested that the AAF in the cat is homologous to the caudomedial (CM) belt area in the primate brain (see de la Mothe et al., 2006 for a discussion of the similarities between the primate CM and cat AAF). Like the primate CM (Recanzone, 2000), both the cat and ferret AAFs have shorter response latencies relative to the A1 and an under‐representation of mid‐frequencies (Imaizumi et al., 2004; Bizley et al., 2005). The AAF in the cat is innervated predominantly by the rostral pole of the MGB and the dorsal division of the MGB, with smaller inputs from the MGBv and MGBm (Imig and Morel, 1983; Morel and Imig, 1987; Lee and Winer, 2008a). Drawing further analogies between the AAF in the ferret and other species therefore awaits detailed investigations of the thalamocortical connectivity.

The ADF shares several properties with the A2 in the cat: both contain neurons that respond to a wide range of frequencies and are not arranged tonotopically, and preliminary investigations have suggested that, like the cat A2, the ADF is not directly innervated by the MGBv. It remains unclear whether these fields are strictly equivalent to one another. As noted above, the aPSSC seems likely to be homologous to the cat fAES.

The posterior fields, PPF and PSF, are both tonotopically organized belt fields and, as such, could be homologous to the PAF and VPAF in the cat. These latter areas are arranged linearly, with the PAF lying ventral to the A1, and the VPAF ventral to the PAF, with tonotopic reversals occurring between the A1 and PAF and again between the PAF and VPAF (Reale and Imig, 1980). In the ferret, in which the frequency gradient of the A1 is typically rotated through 90° relative to that in the cat, both posterior fields occupy the same dorsoventral location and reverse across a common low‐frequency border, both with each other and with the primary fields. It is therefore not immediately clear which fields should be considered as homologous across these species based on the direction of the tonotopic gradients. The responses of neurons in the PAF are often nonmonotonic with respect to sound intensity and have longer latencies and more sustained firing patterns (Stecker et al., 2005), whereas the VPAF response properties remain relatively undocumented. These properties of PAF neurons resemble those of neurons in both the PPF and PSF in the ferret (Bizley et al., 2005).

Anatomically, the feline PAF and VPAF share many properties. Both are innervated by the A1, AAF, A2, and dorsal zone (DZ) of the auditory cortex, although the projection from the AAF and DZ is stronger to the PAF than to the VPAF (Rouiller et al., 1991; Imig and Reale, 1980). The PAF is innervated by the dorsal suprageniculate nucleus, as well as the ventral and dorsal MGB divisions (Lee and Winer, 2008a). Injections in the cat VPAF label projections originating in the caudal MGBv, as well as the laterodorsal nucleus, ventrolateral nucleus, and MGBm (Lee and Winer, 2008a). Projections from the PAF and VPAF are largely similar, connecting strongly with other tonotopic fields. One distinguishing feature is that the VPAF has stronger connections with nontonotopic multisensory areas on the posterior ectosylvian gyrus (Lee and Winer, 2008b).

In common with the cat PAF and VPAF, we found the PPF and PSF to be strongly connected with the A1 while also receiving a smaller input from the AAF. Injections into the PSF, but not the PPF, revealed strong projections to the sulcal region caudal to the PSF. Preliminary data in the ferret suggest that the PPF receives inputs from the MGBv, as well as other MGB subdivisions, but how this compares to the PSF awaits further study. Importantly, the cat PAF and VPAF are functionally distinguishable — sound localization accuracy is impaired after cooling the PAF, whereas this is not the case when the VPAF is deactivated (Malhotra and Lomber, 2007). More persistent pharmacological inactivation of the PEG in ferrets produces a small deficit in sound localization accuracy and disrupts the ability of animals to adapt with training to an asymmetric hearing loss (Nodal et al., 2012), but no attempt has so far been made to distinguish between the effects of silencing neurons in the PSF and PPF.

The primate auditory cortex is organized as a central core of tonotopic areas, surrounded by a belt of nontonotopic belt areas (Hackett et al., 1998: Kaas and Hackett, 1998; de la Mothe et al., 2006). Core areas contain a primary field (the A1) and two tonotopically organized rostral fields (the R and RT). As noted above, the CM in the primate brain has been likened to the AAF in the cat (de la Mothe et al., 2006); if this were the case, then based on physiological response properties, fields R and RT could be considered to be analogous to the ferret PPF and PSF (or the PAF and VPAF in the cat). The nontonotopic areas ADF, VP, and AVF, and the PSSC in the ferret might then be described as higher level fields, perhaps equivalent to primate belt and parabelt areas.

Common principles of cortical organization are also observed in other mammalian species including bats (Esser and Eiermann, 1999; Hoffmann et al., 2008), gerbils (Budinger et al., 2000), guinea pigs (Wallace et al., 2002), rats (Polley et al., 2007; Storace et al., 2010), and mice (Hofstetter and Ehret, 1992; Hackett et al., 2011). Like the ferret, each of these species has a number of tonotopic areas, which are highly interconnected and which receive distinct patterns of thalamic input. Nevertheless, which of these fields are homologous remains unknown.

### Callosal connectivity

Our tracer injections in the MEG reproduced patterns of callosal connectivity that have previously been reported in ferrets (Wallace and Bajwa, 1991; Pallas and Sur, 1993; Wallace and Harper, 1997). Generally, contralateral labeling formed a reduced, but mirrored, pattern to that observed ipsilateral to the injection site. Nevertheless, because we used relatively small deposits of tracer, the callosal labeling was often sparse. A full appreciation of the complexities of callosal labeling frequently observed in the auditory cortex (Hackett and Philipps, 2011), for each of the cortical fields under consideration here, requires additional experiments utilizing larger deposits of tracer.

## CONCLUSIONS

In conclusion, the patterns of cortico‐cortical connectivity we observed suggest that signals are both integrated and segregated as they pass through the auditory cortex. Differences in the connectivity patterns between anterior and posterior areas are consistent with the presence of functionally distinct processing streams. Given the specificity with which visual cortical fields innervate the auditory cortex (Bizley et al., 2007a), it seems likely that, as in the monkey (Hackett et al., 1999; Romanski et al., 1999), distinct differences may exist in the projections from the ferret auditory cortex to prefrontal and parietal areas, although this remains to be tested. Finally, the convergence and divergence of connections found throughout the auditory cortex suggest that behaviorally relevant information can be processed in parallel, providing a potential substrate for compensatory plasticity when specific cortical fields are removed or inactivated for prolonged periods.

## CONFLICT OF INTEREST STATEMENT

The authors declare they have no competing financial interests.

## ROLE OF AUTHORS

All authors had full access to all the data in the study and take responsibility for the integrity of the data and the accuracy of the data analysis. Study concept and design: all authors. Acquisition of data: JKB, VMB, FRN. Analysis and interpretation of data: JKB, VMB, FRN. Drafting of the manuscript: JKB. Critical revision of the manuscript for important intellectual content: VMB, FRN, AJK. Statistical analysis: . Obtained funding: AJK.
